# A Whole-Blood Point-of-Care Test for Highly Specific Serodiagnosis of Human Cysticercosis

**DOI:** 10.3390/pathogens15040399

**Published:** 2026-04-07

**Authors:** Lakkhana Sadaow, Patcharaporn Boonroumkaew, Rutchanee Rodpai, Oranuch Sanpool, Tongjit Thanchomnang, Marcello Otake Sato, Pewpan M. Intapan, Hiroshi Yamasaki, Yasuhito Sako, Toni Wandra, Kadek Swastika, Wanchai Maleewong

**Affiliations:** 1 Mekong Health Science Research Institute, Khon Kaen University, Khon Kaen 40002, Thailand; lakkhasa@kku.ac.th (L.S.); hamooploy@gmail.com (P.B.); rutcro@kku.ac.th (R.R.); oransa@kku.ac.th (O.S.); tongjit.t@msu.ac.th (T.T.); pewpan@kku.ac.th (P.M.I.); 2 Department of Parasitology, Faculty of Medicine, Khon Kaen University, Khon Kaen 40002, Thailand; 3 Department of Parasitology, Faculty of Medicine, Mahasarakham University, Maha Sarakham 44000, Thailand; 4 Division of Global Environment Parasitology, Faculty of Medical Technology, Niigata University of Pharmacy and Medical and Life Sciences, Niigata City 956-8603, Japan; marcello@nupals.ac.jp; 5 Department of Parasitology, National Institute of Infectious Diseases, Japan Institute for Health Security, Shinjuku-ku 162-8640, Japan; hyamasaki2015@yahoo.co.jp; 6 Division of Parasitology, Department of Infectious Diseases, Asahikawa Medical University, Asahikawa 078-8510, Japan; 7 Directorate of Postgraduate, Sari Mutiara Indonesia University, Kota Medan 20123, Indonesia; t_wandra@yahoo.co.id; 8 Department of Parasitology, Faculty of Medicine, Udayana University, Kota Denpasar 80232, Indonesia; kadek_swastika@unud.ac.id

**Keywords:** *Taenia solium*, human cysticercosis, diagnostics, diagnosis, antibody detection, immunochromatographic test, point-of-care test, zoonosis

## Abstract

**Background:** Human cysticercosis, caused by the larval stage (cysticerci) of the pork tapeworm *Taenia solium*, is an important zoonotic disease. The disease is prevalent in developing countries where porcine cysticercosis is common and undercooked pork is habitually consumed. **Objective:** This study aimed to develop an immunochromatography-based test kit for the rapid diagnosis of human cysticercosis using low-molecular-weight antigens purified from cyst fluid of the *T. solium* Asian genotype to detect specific IgG antibodies in whole blood. The kit was designated as “the cysticercosis whole-blood test kit (iCys WB kit).” **Methods:** It was evaluated under laboratory conditions using 164 whole-blood samples, of which 21 were from confirmed cysticercosis cases. The results of the iCys WB kit, which detects anti-*T. solium* (cysticercus) antibodies in simulated whole blood samples, were compared with results from corresponding human serum samples. **Results:** When using both sample types, iCys WB kit demonstrated an accuracy of 98.8%, a sensitivity of 91.7%, a specificity of 100%, a positive likelihood ratio of 0, a negative likelihood ratio of 0.083, and an ROC area of 0.96. The agreement between results obtained from simulated whole-blood and serum samples showed perfect concordance. **Conclusions:** The iCys WB kit is a valuable easy-to-handle diagnostic tool and may be applicable for supporting clinical diagnosis at the point of care.

## 1. Introduction

Cysticercosis is a foodborne zoonosis caused by larvae (cysticerci) of the pork tapeworm, *Taenia solium*. It occurs predominantly in developing countries, affecting people from low-income backgrounds, and is exacerbated by sociocultural practices such as consumption of infected pork, open defecation, free-roaming pigs, and unregulated pig husbandry and slaughterhouses. The disease is endemic to regions where both porcine cysticercosis is widespread and the consumption of undercooked pork is commonplace, primarily in Southeast and South Asia, Sub-Saharan Africa, and Central and South America [1]. Imported cases occasionally appear in non-endemic regions due to international travel [1,2,3]. Taeniasis and cysticercosis are focused on in the Neglected Tropical Diseases Roadmap 2021–2030 for control of public health problems through strategies including preventive chemotherapy, clinical management of taeniasis and neurocysticercosis (NCC), safe and sanitary procedures for the disposal of human fecal matter, improved health and hygiene standards for food safety, prevention of pig access to human feces, pig vaccination and deworming medication, and public health awareness programs [1].

Humans and pigs are essential to the life cycle of *T. solium*, with humans acting as the definitive host and pigs serving as the intermediate host [4]. After consuming undercooked pork that contains cysticerci, adult worms establish themselves and mature within the human intestinal tract. In contrast, when *T. solium* eggs are swallowed, the oncospheres emerge and hatch within the intestine, subsequently traveling through the bloodstream and lymphatic system before ultimately settling and developing into cysticerci within the central nervous system and muscle tissues [4]. Thus, humans can also serve as dead-end intermediate hosts. Occasionally, eggs from intestinal adult worms can hatch within the same host (autoinfection), liberating oncospheres that migrate into tissues and give rise to cysticerci [5].

The invasion of the central nervous system by *T. solium* cysticerci leads to NCC, which commonly manifests as neurological disorders such as epileptic seizures and paralysis [6]. Significantly, NCC is recognized as the foremost cause of epilepsy, accounting for 30% of cases in rural communities where humans and free-roaming pigs live in close contact [1]. Other forms of cysticercosis include subcutaneous cysticercosis, characterized by immobile nodules in the musculature (including the extremities), and ocular/orbital cysticercosis, in which the eyes are infested [7].

Precise diagnosis of human cysticercosis is crucial for appropriate treatment and the prevention of serious clinical manifestations [8]. Diagnosis typically relies on neuroimaging using computed tomography and magnetic resonance imaging, combined with serological and pathological findings [9]. Various serodiagnostic tools, such as ELISA and immunoblot assays, utilize antigens derived from crude or partially purified *T. solium* cyst fluid or cyst-tissue extracts [4]. Recombinant antigens [10] and peptide antigens [11] have also been employed. Nonetheless, these serological techniques are characterized by their time-consuming nature, high cost, and dependence on sophisticated equipment, infrastructure, and specialized technicians, limiting their applicability in resource-poor settings.

Recently, we developed an immunogold-labeled immunochromatography test (ICT) called the “iCysticercosis kit” as a point-of-care test (POCT) for the detection of anti-*T. solium* IgG antibodies within human serum specimens [12]. This kit used crude cyst fluid from Brazilian *T. solium* isolates as the antigen source, demonstrating high diagnostic values [12]. We subsequently attempted to improve the diagnostic specificity using low-molecular-weight antigens (LMWAgs) purified from *T. solium* cyst fluids of different geographical isolates (Latin American and Asian genotypes) [13]. No statistically significant difference in diagnostic values was observed between purified antigens from different *T. solium* genotypes, though specificities were superior to those of the previously reported ICT kit using crude antigen [12]. The previous kits we developed were optimized to detect specific IgG antibodies against *T. solium* in serum samples. Therefore, in this study, we used purified LMWAgs derived from the Asian genotype of *T. solium* [13] and optimized the assay for the detection of specific IgG antibodies in whole-blood samples (WBS) rather than serum.

## 2. Materials and Methods

### 2.1. Preparation of LMWAgs

The antigen platform used in this study was the same as that previously described [13]. *Taenia solium* cysticerci (Asian genotype) were collected from necropsied pigs in Bali, Indonesia, and confirmed by mitochondrial cytochrome *c* oxidase subunit I gene sequencing [14]. The LMWAgs were prepared using the established methods [13,15].

### 2.2. Validation of the Diagnostic Kit for Whole-Blood Applications

We analyzed frozen serum samples (*n* = 164) from the serum banks of the Faculty of Medicine and Mekong Health Science Research Institute Biobank, Khon Kaen University, Thailand, and the Department of Parasitology, National Institute of Infectious Diseases, Tokyo, Japan (Table 1). The sample bank used in this study was the same as that previously described [13]. Nearly all sera were from Asian individuals. Demographics and diagnostic criteria for *T. solium* cysticercosis patients (*n* = 24) were previously described [12]. The non-cysticercosis sera (Table 1) have been previously described [12], with the exception of the loiasis (*n* = 2) and anisakiasis (*n* = 5) samples [16].

This study was conducted per the Declaration of Helsinki and approved by the Center for Ethics in Human Research at Khon Kaen University (HE664044) and the Medical Ethics Committee of the National Institute of Infectious Diseases, Tokyo, Japan (Nos. 177, 589). The Ethics Committee waived informed consent requirements. All samples were coded and fully anonymized.

All 164 serum samples were used to prepare WBSs by spiking with red blood cells (RBCs), as previously described [13,17].

### 2.3. Immunochromatographic Test Kit Named “iCys WB Kit”

For ICT kit production, LMWAgs (I2) were used as the antigen to detect total IgG antibodies. The kit components have been described previously [13]. The reproducibility and consistency of the kit was stable for 24 months at ambient temperature (4–30 °C).

**Figure 1:**
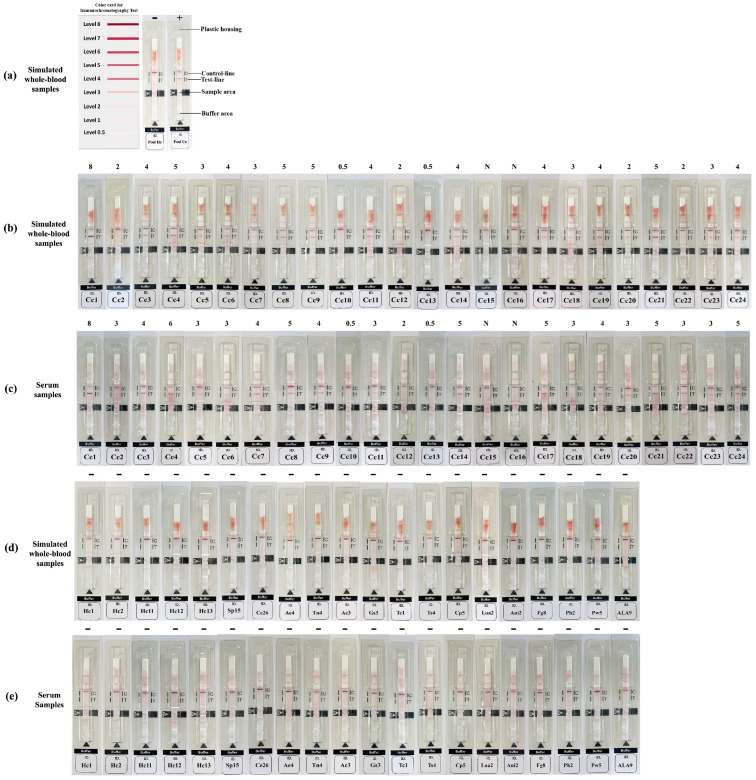
Representative examples of anti-*T. solium* antibody detection in simulated whole-blood and serum using the iCys WB kit. Positive and negative examples and reference card board (**a**), and reactions with simulated whole-blood (**b**,**d**) and the corresponding serum samples (**c**,**e**). Cc1–Cc24, cysticercosis; Hc1–Hc2 and Hc11–Hc13, healthy Thai and Japanese controls, respectively; Sp15, sparganosis; Ce26, cystic echinococcosis; Ae4, alveolar echinococcosis; Tn4, taeniasis (*Taenia saginata*); Ac3, angiostrongyliasis; Gs3, gnathostomiasis; Tc1, toxocariasis; Ts4, trichinosis; Cp5, capillariasis (*Capillaria philippinensis*); Loa2, loiasis; Ani2, anisakiasis; Fg8, fascioliasis (*Fasciola gigantiga*)*;* Ph2 paragonimiasis (*Paragonimus heterotremus*); Pw5, paragonimiasis (*Paragonimus westermani*); ALA9, amoebiasis. The color-intensity levels (0.5–8) are shown above each strip. N or - indicates negative results.

To perform the test, WBS or serum samples were diluted 1:5 with chromatography buffer. A 5 µL drop of diluted sample was applied to the sample (S) area, followed by 60 µL of chromatography buffer to the buffer area (Figure 1a). Results were interpreted after 15 min based on the appearance of red bands. Two red bands appear at both test (T) and control (C) lines, indicating that it is positive, while a red band appears at C line, indicating that it is negative. If no band appears at C-line, it is an invalid assay. Positive band intensity (test-line) was visually compared with a reference color card (minimum cutoff: 0.5) (Figure 1a).

Data analysis was performed using STATA version 10.1 (StataCorp LLC., College Station, TX, USA). Sensitivity, specificity, and cross-reactivity were compared using McNemar’s test. Overall concordance was calculated using Cohen’s kappa test, with κ values interpreted as almost perfect agreement [18]. This study followed STARD 2015 criteria for reporting diagnostic accuracy [19].

## 3. Results

The results of the iCys WB kit, which detects anti-*T. solium* (cysticercus) antibodies in simulated WBS and corresponding human serum samples, are presented in Figure 1, Table 1, and Supplementary Table S1. Twenty-two out of 24 cysticercosis samples were positive, while none of the samples from healthy persons and other parasitosis groups were positive. When using both sample types, the iCys WB kit demonstrated an accuracy of 98.8% [95% CI: 95.7–99.8%], a sensitivity of 91.7% [95% CI: 73.0–99.0%], a specificity of 100% [95% CI: 97.4–100.0%], a positive likelihood ratio of 0, a negative likelihood ratio of 0.083 [95% CI: 0.021–0.314], and an ROC area of 0.96 [95% CI: 0.90–1.00]. The agreement between results, obtained from WBS and serum samples, was further evaluated using Cohen’s kappa, which yielded a value of 1.0, indicating perfect agreement with an overall concordance of 100%.

## 4. Discussion

Currently, serodiagnosis of cysticercosis using the chromatographic lateral-flow approach as a POCT tool is only available with recombinant proteins [20,21] or native *T. solium* cyst-fluid antigen [12]. The former POCT kit (the TS POC kit, which uses recombinant rES33 protein antigen) demonstrated a sensitivity of 88–93% and a specificity of 99%. The latter POCT kit (the iCysticercosis kit, which uses *T. solium* cyst-fluid antigen) showed high sensitivity (83.3%) and specificity (92.0%) in detecting cysticercosis; however, it produced numerous cross-reactions with human cystic echinococcosis (33.3%) and alveolar echinococcosis (16.7%) [12]. This cross-reactivity may be attributed to echinococcosis patients, particularly those with cystic echinococcosis, producing antibodies to *Echinococcus* Antigen B, which is a member of the same protein family as the LMWAgs of *T. solium* [22,23]. Sadaow et al. [13] recently evaluated the diagnostic performance of ICT kits featuring LMWAgs, purified from the cyst fluids of American (B1) and Asian (I2) *T. solium* genotypes. The sensitivity, specificity, and accuracy were 83.3%, 93.6%, and 92.1%, respectively for the American genotype-based ICT kit, while for the Asian genotype-based ICT kit, the sensitivity, specificity, and accuracy were 87.5%, 98.6%, and 97.0%, respectively (Table 2). The LMWAgs based-ICT tests showed superior immunodiagnostic effectiveness using *T. solium* cyst fluids, with remarkable specificity and sensitivity for accurately distinguishing and serologically diagnosing NCC, as well as for markedly reduced cross-reactivity [13].

In this study, we optimized the previous ICT kit to detect IgG antibodies in the whole-blood samples instead of serum samples using LMWAgs purified from cyst fluids of *T. solium* Asian genotype, evaluated their diagnostic performance in simulated WBSs, and compared their effectiveness with matching serum samples. Our approach was grounded in the finding that cross-reactive responses with echinococcosis that had been detected in ICT-based serological diagnosis when utilizing *T. solium* cyst fluid [12] are reduced when using cation-exchange chromatography-purified LMWAgs without compromising sensitivity for cysticercosis [13]. Here, we successfully developed a new ICT kit with higher sensitivity (91.7%) than the previous ICT kit (iCysticercosis kit) [12] as well as both the American genotype-based ICT kit and the Asian genotype-based ICT kit [13], while achieving reduced cross-reactivity (100% specificity). Although one case each of cystic echinococcosis and toxocariasis cross-reacted with the Asian genotype-based ICT kit [13], no cross-reactions were observed with the present iCys WB kit. These results are likely due to different optimal conditions, such as serum dilution and buffer types.

A key finding was that no false-positive reactions occurred in patients with parasitic diseases that can be difficult to distinguish from cysticercosis, including cerebral sparganosis, paragonimiasis westermani, cystic echinococcosis, alveolar echinococcosis, and amoebiasis. The kit may be employed as a practical tool to aid in clinical diagnosis at the point of care, as well as to facilitate large-scale sero-epidemiological studies in remote endemic locations where medical facilities and supplementary supplies may not be readily available. Additionally, this POCT kit can potentially be used with fingerprick blood samples, eliminating the need for venous blood collection and serum separation.

To date, clinicians and laboratory technologists should recognize that the assessment of the cysticercosis whole-blood test kit has been restricted to laboratory conditions, utilizing only a small and defined set of samples. The kit’s performance may be affected by possible cross-reactivity with other diseases in the regions where it could ultimately be used. As a result, further evaluations are needed using a wider variety of samples from different parasitic infections, as well as from real-world populations, given that our study relied on archived frozen sera. Diagnostic accuracy may vary depending on the population tested. To that end, we are currently coordinating a pilot study in the regions where cysticercosis is prevalent, such as Indonesia, among others.

In our study, purified LMWAgs were obtained from native cysticerci. To improve the kit, future research should evaluate new, highly sensitive and specific recombinant antigens [24]. Such enhancements would ensure a stable antigen supply, reduce the costs associated with native cysticerci collection, simplify quality control, and likely improve the diagnostic performance of the test.

## 5. Conclusions

The present work proposes a cysticercosis whole-blood test kit using purified antigens derived from *T. solium*. This kit yielded no statistically relevant difference in the diagnostic parameters for cysticercosis diagnosis between simulated WBSs and serum samples. When compared to a former ICT kit that incorporated crude antigen, these diagnostic values proved to be higher (Table 2). The POCT kit may be applicable for both bedside and field use.

## Figures and Tables

**Table 1:** Evaluation of the iCys WB kit using simulated whole-blood and serum samples.

Category of Serum Samples	Number Positive/Total (Range of Band Intensity Levels)	
	Simulated Whole-Blood Samples	Serum Samples
Healthy persons	0/30 (0)	0/30 (0)
Cysticercosis (*Taenia solium*)	22/24 (0.5–8)	22/24 (0.5–8)
Sparganosis	0/12 (0)	0/12 (0)
Cystic echinococcosis	0/28 (0)	0/28 (0)
Alveolar echinococcosis	0/6 (0)	0/6 (0)
Taeniasis (*Taenia saginata*)	0/5 (0)	0/5 (0)
Angiostrongyliasis (Eosinophilic meningitis)	0/10 (0)	0/10 (0)
Gnathostomiasis	0/5 (0)	0/5 (0)
Toxocariasis	0/2 (0)	0/2 (0)
Trichinosis	0/5 (0)	0/5 (0)
Capillariasis	0/5 (0)	0/5 (0)
Loiasis	0/2 (0)	0/2 (0)
Anisakiasis	0/5 (0)	0/5 (0)
Fascioliasis (*Fasciola gigantica*)	0/10 (0)	0/10 (0)
Paragonimiasis *	0/10 (0)	0/10 (0)
Amoebiasis	0/5 (0)	0/5 (0)
Sensitivity (%)	91.7 (95% CI [73.0–99.0])	91.7 (95% CI [73.0–99.0])
Specificity (%)	100.0 (95% CI [97.4–100.0])	100.0 (95% CI [97.4–100.0])
Positive likelihood ratio	0	0
Negative likelihood ratio	0.08 (95% CI [0.02–0.31])	0.08 (95% CI [0.02–0.31])
Accuracy (%)	98.8 (95% CI [95.6–99.8])	98.8 (95% CI [95.6–99.8])

* *Paragonimus heterotremus* and *P. westermani*.

**Table 2:** Comparison of diagnostic performance of chromatographic lateral-flow approach by previous and present reports.

Authors	Sample Types	Antigens	Sensitivity (%)	Specificity (%)	Accuracy (%)
Sadaow et al., 2023 [12]	Sera	Native crude cyst fluid (*Taenia solium* American genotype)	83.3	92.0	90.9
Sadaow et al., 2026 [13]	Sera	Purified LMWAgs (*T. solium* American and Asian genotypes)	83.3–87.5	93.6–98.6	92.1–97.0
Mubanga et al., 2021 [20]	Blood	Recombinant T24H (rT24H) protein	88–93	99	Not available
The present study	Sera or Blood	Purified LMWAg (*T. solium* Asian genotype)	91.7	100	98.8

## Data Availability

The original contributions presented in this study are included in the article. Further inquiries can be directed to the corresponding author.

## Supplementary Materials

The following supporting information can be downloaded at: https://www.mdpi.com/article/10.3390/pathogens15040399/s1. Table S1: Diagnostic performance of specific IgG antibody detection in simulated whole blood and corresponding serum samples using a cysticercosis immunochromatographic test tool.

## Abbreviations

The following abbreviations are used in this manuscript:

EDTA	Ethylenediaminetetraacetic acid
IgG	Immunoglobulin G
ROC	Receiver Operating Characteristic
NCC	Neurocysticercosis
ELISA	Enzyme-Linked Immunosorbent Assay
POCT	Point-of-Care Test
LMWAgs	Low-Molecular-Weight Antigens
ICT	Immunochromatographic Test
WBSs	Whole-Blood Samples
95% CI	95% Confidence Interval
