# Supplementary material for: A Whole-Blood Point-of-Care Test for Highly Specific Serodiagnosis of Human Cysticercosis
Source: Pathogens. 2026 Apr 7;15(4):399. doi: 10.3390/pathogens15040399 (PMC13118316; doi:10.3390/pathogens15040399)
Supplement: Supplementary file 1 [file pathogens-15-00399-s001.zip › pathogens-4161656-supplementary.pdf]

Table S1. Diagnostic performance of specific IgG antibody detection in simulated whole blood and corresponding serum samples using a cysticercosis immunochromatographic test tool.

No.	Case nos.	Category of samples	Diagnostic criteria	Suspected origin of infection	ICT results intensity of positive band	
					Simulated Whole Blood	Serum
1	Hc1	Healthy persons	FECT and serology	Thailand	0	0
2	Hc2	Healthy persons	FECT and serology	Thailand	0	0
3	Hc3	Healthy persons	FECT and serology	Thailand	0	0
4	Hc4	Healthy persons	FECT and serology	Thailand	0	0
5	Hc5	Healthy persons	FECT and serology	Thailand	0	0
6	Hc6	Healthy persons	FECT and serology	Thailand	0	0
7	Hc7	Healthy persons	FECT and serology	Thailand	0	0
8	Hc8	Healthy persons	FECT and serology	Thailand	0	0
9	Hc9	Healthy persons	FECT and serology	Thailand	0	0
10	Hc10	Healthy persons	FECT and serology	Thailand	0	0
11	Hc11	Healthy persons	FECT and serology	Japan	0	0
12	Hc12	Healthy persons	FECT and serology	Japan	0	0
13	Hc13	Healthy persons	FECT and serology	Japan	0	0
14	Hc14	Healthy persons	FECT and serology	Japan	0	0
15	Hc15	Healthy persons	FECT and serology	Japan	0	0
16	Hc16	Healthy persons	FECT and serology	Japan	0	0
17	Hc17	Healthy persons	FECT and serology	Japan	0	0
18	Hc18	Healthy persons	FECT and serology	Japan	0	0
19	Hc19	Healthy persons	FECT and serology	Japan	0	0
20	Hc20	Healthy persons	FECT and serology	Japan	0	0
21	Hc21	Healthy persons	FECT and serology	Japan	0	0
22	Hc22	Healthy persons	FECT and serology	Japan	0	0
23	Hc23	Healthy persons	FECT and serology	Japan	0	0
24	Hc24	Healthy persons	FECT and serology	Japan	0	0
25	Hc25	Healthy persons	FECT and serology	Japan	0	0
26	Hc26	Healthy persons	FECT and serology	Japan	0	0
27	Hc27	Healthy persons	FECT and serology	Japan	0	0
28	Hc28	Healthy persons	FECT and serology	Japan	0	0
29	Hc29	Healthy persons	FECT and serology	Japan	0	0
30	Hc30	Healthy persons	FECT and serology	Japan	0	0
31	Cc1	NCC (racemose-type)	CT, MRI, Serology, pathology	China	8	8

No.	Case nos.	Category of samples	Diagnostic criteria	Suspected origin of infection	ICT results intensity of positive band	
					Simulated Whole Blood	Serum
32	Cc2	NCC (multiple)	CT, MRI, PET, Serology	Malawi	2	3
33	Cc3	Ocular cysticercosis and taeniasis	Fundoscopy, US, Serology	Thailand, Laos, or Madagascar?	4	4
34	Cc4	NCC (multiple), T. solium taeniasis	CT, MRI, Serology, DNA analysis, Capsule endoscopy for taeniasis	India	5	6
35	Cc5	NCC (multiple, racemose type)	CT, MRI, US, Serology, Pathology, DNA analysis	Japan	3	3
36	Cc6	NCC (racemose-type)	CT, MRI, Serology, Pathology, DNA analysis	India	4	3
37	Cc7	NCC (multiple), SCC (multiple)	CT, MRI, US, Serology	China	3	4
38	Cc8	NCC (multiple), SCC (multiple) and T. solium taeniasis	CT, MRI, Serology, DNA analysis	India	5	5
39	Cc9	NCC (multiple), SCC (multiple)	CT, Serology	India	5	4
40	Cc10	SCC (solitary)	CT, US, Serology, Pathology, DNA analysis	Nepal	0.5	0.5
41	Cc11	NCC (solitary)	Serology	India	4	3
42	Cc12	NCC (multiple), SCC (multiple)	CT, MRI, Serology, Pathology	Cambodia	2	2
43	Cc13	NCC (multiple)	Serology	Nepal	0.5+	0.5
44	Cc14	NCC (spinal, solitary)	Serology, Pathology, DNA analysis	Brazil	4	5
45	Cc15	NCC (multiple), SCC (multiple)	CT, MRI, Serology, DNA analysis	India	0	0
46	Cc16	NCC (multiple)	CT, MRI, DNA analysis, serology	Nepal	0	0
47	Cc17	Ocular cysticercosis	Surgical operation and removal of eye	Thailand	4	5
48	Cc18	NCC (multiple), SCC (multiple)	CT, MRI	Thailand	3	3
49	Cc19	NCC	CT	Thailand	4	4
50	Cc20	NCC (multiple), SCC (left arm)	X-ray, Pathology	Thailand	2	3
51	Cc21	NCC (multiple)	CT, Serology	Thailand	5	5
52	Cc22	NCC (multiple)	MRI, Serology	Thailand	2	3
53	Cc23	NCC (multiple)	CT, Serology	Thailand	3	3
54	Cc24	NCC (multiple)	Biopsy, Pathology	Thailand	4	5
55	Sp1	Sparganosis	Serology	Thailand	0	0
56	Sp2	Sparganosis	Pathology	Thailand	0	0
57	Sp3	Sparganosis	Serology	Thailand	0	0
58	Sp4	Sparganosis	Pathology	Thailand	0	0
59	Sp5	Sparganosis	Pathology	Thailand	0	0
60	Sp6	Sparganosis (cerebral type, proven case)	CT, MRI, pathology, serology	Japan	0	0

No.	Case nos.	Category of samples	Diagnostic criteria	Suspected origin of infection	ICT results intensity of positive band	
					Simulated Whole Blood	Serum
61	Sp7	Sparganosis and spirometrosis (proven case)	CT, MRI, Pathology, Serology	Japan	0	0
62	Sp8	Sparganosis (*Spirometra mansoni*)	Surgery, pathology, serology, DNA	Japan	0	0
63	Sp9	Sparganosis (proven case)	MRI serology, pathology	Japan	0	0
64	Sp10	subcutaneous sparganosis, (*Spirometra mansoni*)	CT, MRI, serology, DNA	Japan	0	0
65	Sp11	Sparganosis	Serology	Japan	0	0
66	Sp12	Sparganosis	Serology	Japan	0	0
67	Ce1	Cystic echinococcosis	Ultrasonography and serology	unknown	0	0
68	Ce2	Cystic echinococcosis	Ultrasonography and serology	Peru	0	0
69	Ce3	Cystic echinococcosis	Ultrasonography and serology	Afghanistan	0	0
70	Ce4	Cystic echinococcosis	Ultrasonography and serology	Nepal	0	0
71	Ce5	Cystic echinococcosis	Ultrasonography and serology	China	0	0
72	Ce6	Cystic echinococcosis	Ultrasonography and serology	China	0	0
73	Ce7	Cystic echinococcosis	Ultrasonography and serology	China	0	0
74	Ce8	Cystic echinococcosis	Ultrasonography and serology	China	0	0
75	Ce9	Cystic echinococcosis	Ultrasonography and serology	China	0	0
76	Ce10	Cystic echinococcosis	Ultrasonography and serology	China	0	0
77	Ce11	Cystic echinococcosis	Ultrasonography and serology	China	0	0
78	Ce12	Cystic echinococcosis	Ultrasonography and serology	China	0	0
79	Ce13	Cystic echinococcosis	Ultrasonography and serology	China	0	0
80	Ce14	Cystic echinococcosis	Ultrasonography and serology	China	0	0
81	Ce15	Cystic echinococcosis	Ultrasonography and serology	China	0	0
82	Ce16	Cystic echinococcosis	Ultrasonography and serology	China	0	0
83	Ce17	Cystic echinococcosis	Ultrasonography and serology	China	0	0
84	Ce18	Cystic echinococcosis	Ultrasonography and serology	China	0	0
85	Ce19	Cystic echinococcosis	Ultrasonography and serology	China	0	0
86	Ce20	Cystic echinococcosis	Ultrasonography and serology	China	0	0
87	Ce21	Cystic echinococcosis	Ultrasonography and serology	China	0	0
88	Ce22	Cystic echinococcosis	Ultrasonography and serology	China	0	0
89	Ce23	Cystic echinococcosis	Ultrasonography and serology	China	0	0
90	Ce24	Cystic echinococcosis	Ultrasonography and serology	China	0	0
91	Ce25	Cystic echinococcosis	Ultrasonography and serology	China	0	0
92	Ce26	Cystic echinococcosis	Ultrasonography and serology	China	0	0

No.	Case nos.	Category of samples	Diagnostic criteria	Suspected origin of infection	ICT results intensity of positive band	
					Simulated Whole Blood	Serum
93	Ce27	Cystic echinococcosis	Ultrasonography and serology	China	0	0
94	Ce28	Cystic echinococcosis	Ultrasonography and serology	China	0	0
95	Ae1	Alveolar echinococcosis	Ultrasonography and serology	Japan	0	0
96	Ae2	Alveolar echinococcosis	Ultrasonography and serology	Japan	0	0
97	Ae3	Alveolar echinococcosis	Ultrasonography and serology	China	0	0
98	Ae4	Alveolar echinococcosis	Ultrasonography and serology	China	0	0
99	Ae5	Alveolar echinococcosis	Ultrasonography and serology	China	0	0
100	Ae6	Alveolar echinococcosis	Ultrasonography and serology	China	0	0
101	Tn1	Taeniasis (*Taenia saginata*)	FECT	Thailand	0	0
102	Tn2	Taeniasis (*Taenia saginata*)	FECT	Thailand	0	0
103	Tn3	Taeniasis (*Taenia saginata*)	FECT	Thailand	0	0
104	Tn4	Taeniasis (*Taenia saginata*)	FECT	Thailand	0	0
105	Tn5	Taeniasis (*Taenia saginata*)	FECT	Thailand	0	0
106	Ac1	Angiostrongyliasis (ocular)	Worm removal and Serology	Thailand	0	0
107	Ac2	Angiostrongyliasis (ocular)	Worm removal and Serology	Thailand	0	0
108	Ac3	Angiostrongyliasis (ocular)	Worm removal and Serology	Thailand	0	0
109	Ac4	Angiostrongyliasis (ocular)	Worm removal and Serology	Thailand	0	0
110	Ac5	Angiostrongyliasis (eosinophilic meningitis)	Serology	Thailand	0	0
111	Ac6	Angiostrongyliasis (eosinophilic meningitis)	Serology	Thailand	0	0
112	Ac7	Angiostrongyliasis (eosinophilic meningitis)	Serology	Thailand	0	0
113	Ac8	Angiostrongyliasis (eosinophilic meningitis)	Serology	Thailand	0	0
114	Ac9	Angiostrongyliasis (eosinophilic meningitis)	Serology	Thailand	0	0
115	Ac10	Angiostrongyliasis (eosinophilic meningitis)	Serology	Thailand	0	0
116	Gn1	Gnathostomiasis	Serology	Thailand	0	0
117	Gn2	Gnathostomiasis	Serology	Thailand	0	0
118	Gn3	Gnathostomiasis	Serology	Thailand	0	0
119	Gn4	Gnathostomiasis	Serology	Thailand	0	0
120	Gn5	Gnathostomiasis	Serology	Thailand	0	0
121	Tc1	Toxocariasis	Serology	Japan	0	0
122	Tc2	Toxocariasis	Serology	Japan	0	0
123	Ts1	Trichinosis	Serology	Thailand	0	0
124	Ts2	Trichinosis	Serology	Thailand	0	0
125	Ts3	Trichinosis	Serology	Thailand	0	0

No.	Case nos.	Category of samples	Diagnostic criteria	Suspected origin of infection	ICT results intensity of positive band	
					Simulated Whole Blood	Serum
126	Ts4	Trichinosis	Serology	Thailand	0	0
127	Ts5	Trichinosis	Serology	Thailand	0	0
128	Cp1	Capillariasis	FECT	Thailand	0	0
129	Cp2	Capillariasis	FECT	Thailand	0	0
130	Cp3	Capillariasis	FECT	Thailand	0	0
131	Cp4	Capillariasis	FECT	Thailand	0	0
132	Cp5	Capillariasis	FECT	Thailand	0	0
133	Loa1	Loiasis (Loa loa)	Worm removal	Zaire (currently Democratic Rebublic of the Congo)	0	0
134	Loa2	Loiasis (Loa loa)	Worm removal	Zaire (currently Democratic Rebublic of the Congo)	0	0
135	An1	Anisakiasis	Worm removal	Japan	0	0
136	An2	Anisakiasis	Worm removal	Japan	0	0
137	An3	Anisakiasis	Worm removal	Japan	0	0
138	An4	Anisakiasis	Worm removal	Japan	0	0
139	An5	Anisakiasis	Worm removal	Japan	0	0
140	Fg1	Fascioliasis (*Fasciola gigantica*)	FECT and Serology	Thailand	0	0
141	Fg2	Fascioliasis (*Fasciola gigantica*)	FECT and Serology	Thailand	0	0
142	Fg3	Fascioliasis (*Fasciola gigantica*)	FECT and Serology	Thailand	0	0
143	Fg4	Fascioliasis (*Fasciola gigantica*)	FECT and Serology	Thailand	0	0
144	Fg5	Fascioliasis (*Fasciola gigantica*)	FECT and Serology	Thailand	0	0
145	Fg6	Fascioliasis (*Fasciola gigantica*)	FECT and Serology	Thailand	0	0
146	Fg7	Fascioliasis (*Fasciola gigantica*)	FECT and Serology	Thailand	0	0
147	Fg8	Fascioliasis (*Fasciola gigantica*)	FECT and Serology	Thailand	0	0
148	Fg9	Fascioliasis (*Fasciola gigantica*)	FECT and Serology	Thailand	0	0
149	Fg10	Fascioliasis (*Fasciola gigantica*)	FECT and Serology	Thailand	0	0
150	Ph1	Paragonimiasis (*P. heterotremus*)	Western blot and the presence of eggs in sputa or feces	Thailand	0	0
151	Ph2	Paragonimiasis (*P. heterotremus*)	Western blot and the presence of eggs in sputa or feces	Thailand	0	0
152	Ph3	Paragonimiasis (*P. heterotremus*)	Western blot and the presence of eggs in sputa or feces	Thailand	0	0
153	Ph4	Paragonimiasis (*P. heterotremus*)	Western blot and the presence of eggs in sputa or feces	Thailand	0	0
154	Pw5	Paragonimiasis (cerebral, due to *P. westermani*	Clinical signs and serology	Japan	0	0

No.	Case nos.	Category of samples	Diagnostic criteria	Suspected origin of infection	ICT results intensity of positive band	
					Simulated Whole Blood	Serum
155	Pw6	Paragonimiasis (*P. westermani*)	Clinical signs and serology	Japan	0	0
156	Pw7	Paragonimiasis (*P. westermani*)	Clinical signs and serology	Japan	0	0
157	Pw8	Paragonimiasis (*P. westermani*)	Clinical signs and serology	Japan	0	0
158	Pw9	Paragonimiasis (*P. westermani*)	Clinical signs and serology	Japan	0	0
159	Pw10	Paragonimiasis (*P. westermani*)	Clinical signs and serology	Japan	0	0
160	Am1	Amoebiasis (cerebral abscess)	Ultrasound and CT and serology	Japan	0	0
161	Am2	Amoebiasis (liver abscess)	Ultrasound and CT and serology	Japan	0	0
162	Am3	Amoebiasis (liver abscess)	Ultrasound and CT and serology	Japan	0	0
163	Am4	Amoebiasis (liver abscess)	Ultrasound and CT and serology	Japan	0	0
164	Am5	Amoebiasis (liver abscess)	Ultrasound and CT and serology	Japan	0	0
